# Carboxymethyl chitosan prolongs adenovirus‐mediated expression of IL‐10 and ameliorates hepatic fibrosis in a mouse model

**DOI:** 10.1002/btm2.10306

**Published:** 2022-03-10

**Authors:** Yannian Gou, Yaguang Weng, Qian Chen, Jinghong Wu, Hao Wang, Jiamin Zhong, Yang Bi, Daigui Cao, Piao Zhao, Xiangyu Dong, Meichun Guo, William Wagstaff, Bryce Hendren‐Santiago, Connie Chen, Andrew Youssef, Rex C. Haydon, Hue H. Luu, Russell R. Reid, Le Shen, Tong‐Chuan He, Jiaming Fan

**Affiliations:** ^1^ Ministry of Education Key Laboratory of Diagnostic Medicine, and Department of Clinical Biochemistry, School of Laboratory Medicine Chongqing Medical University Chongqing China; ^2^ Molecular Oncology Laboratory, Department of Orthopaedic Surgery and Rehabilitation Medicine The University of Chicago Medical Center Chicago Illinois USA; ^3^ Health Management Center, Deyang People's Hospital Deyang China; ^4^ Stem Cell Biology and Therapy Laboratory of the Pediatric Research Institute, the National Clinical Research Center for Child Health and Disorders, and Ministry of Education Key Laboratory of Child Development and Disorders The Children's Hospital of Chongqing Medical University Chongqing China; ^5^ Department of Orthopaedic Surgery The Affiliated Hospital of the University of Chinese Academy of Sciences, and Chongqing General Hospital Chongqing China; ^6^ Department of Orthopaedic Surgery The First Affiliated Hospital of Chongqing Medical University Chongqing China; ^7^ Laboratory of Craniofacial Suture Biology and Development, Department of Surgery Section of Plastic Surgery The University of Chicago Medical Center Chicago Illinois USA; ^8^ Department of Surgery The University of Chicago Medical Center Chicago Illinois USA

**Keywords:** adenovirus vector, carboxymethyl chitosan (CMC), chitosan, chitosan, gene delivery, gene therapy, hepatic fibrosis, host immune response

## Abstract

Effective and safe liver‐directed gene therapy has great promise in treating a broad range of liver diseases. While adenoviral (Ad) vectors have been widely used for efficacious in vivo gene delivery, their translational utilities are severely limited due to the short duration of transgene expression and solicitation of host immune response. Used as a promising polymeric vehicle for drug release and nucleic acid delivery, carboxymethyl chitosan (CMC) is biocompatible, biodegradable, anti‐microbial, inexpensive, and easy accessible. Here, by exploiting its biocompatibility, controlled release capability and anti‐inflammatory activity, we investigated whether CMC can overcome the shortcomings of Ad‐mediated gene delivery, hence improving the prospect of Ad applications in gene therapy. We demonstrated that in the presence of optimal concentrations of CMC, Ad‐mediated transgene expression lasted up to 50 days after subcutaneous injection, and at least 7 days after intrahepatic injection. Histologic evaluation and immunohistochemical analysis revealed that CMC effectively alleviated Ad‐induced host immune response. In our proof‐of‐principle experiment using the CCl_4_‐induced experimental mouse model of chronic liver damage, we demonstrated that repeated intrahepatic administrations of Ad‐IL10 mixed with CMC effectively mitigated the development of hepatic fibrosis. Collectively, these results indicate that CMC can improve the prospect of Ad‐mediated gene therapy by diminishing the host immune response while allowing readministration and sustained transgene expression.

## 1 INTRODUCTION

Liver is a key organ in the human body and carries out a variety of essential functions including digestion, metabolism, detoxification, immunity and blood clotting.^1^ At the cellular level, hepatocytes constitute the vast majority of cells in the liver parenchyma and are implicated in the majority of monogenic liver inherited disorders, metabolic disorders, viral infections, and malignancies.^1^, ^2^, ^3^ Even though conventional treatments can alleviate symptoms of some liver disorders, very few curative treatments currently exist.^1^ Therefore, effective and safe liver‐directed gene therapy holds great promise in treating a broad range of liver diseases, such as cancer, metabolic disorders, and certain monogenic disorders^1^, ^2^, ^3^ .

Liver is an ideal target for gene therapy since it is one of the largest organs in the human body, and contains 10%–15% of the total blood volume of the body. For the past several decades, numerous efforts have been devoted to the development of liver‐directed gene delivery systems.^1^, ^2^, ^3^ The gene delivery systems are in general divided into two categories: viral vector‐based and non‐viral vector‐based delivery systems. The viral vector systems include a large group of recombinant, replication‐deficient viruses; and the most commonly‐used ones are adenoviral (Ad) vectors, adeno‐associated viral (AAV) vectors, lentiviral vectors, as well as certain less frequently used ones such as foamy viral vectors and herpes simplex viral vectors.^3^, ^4^, ^5^, ^6^, ^7^, ^8^ While viral vectors are highly effective in gene delivery and have been used in approximately 70% clinical trials, the major hurdles for viral vectors include potential carcinogenesis and/or immunogenicity. Non‐viral vector‐based delivery systems take advantage of receptor‐mediated endocytosis and/or membrane fusion functions via the use of lipids, polymers, proteins, and peptides, as well as by physical forces such as needle injection, gene gun, electroporation, sonoporation, and hydrodynamic delivery. While the major challenge for non‐viral vector‐mediated gene delivery is its relatively low efficiency, an increasing number of non‐viral vectors are emerging as valid vehicles for the delivery of genetic materials, especially the use of lipid‐based nanoparticles for RNA delivery.

Nonetheless, viral vectors remain as one of the most preferred approaches to target hepatocytes. Among them, Ad vector represents the prototype viral vector with high gene transfer efficiency, well‐understood virology and pathogenicity, and ease to mass production.^6^, ^7^ The major drawbacks for Ad vector as a liver‐targeted delivery system are a relatively short‐term of transgene expression and the solicitation of host immune response. Therefore, it is conceivable that overcoming the above shortcomings should make Ad vector a more desirable gene delivery vector for liver‐directed gene therapy.

As a product of the deacetylation of chitin, chitosan is a linear polysaccharide, multi‐functional, and eco‐friendly antifouling polymer.^9^, ^10^, ^11^, ^12^ Chitosan is nontoxic, biocompatible, and biodegradable, which makes it a polymer of choice for many biomedical and pharmaceutical applications since it was first reported in late 1990s.^12^ The chemical versatility of chitosan and its derivatives is reflected by its ability to form a poly‐cationic charged polymer at physiological pH, and by its modifiable molecular weight.^12^ The chemical and biological properties of chitosan are dependent on the degree of deacetylation, polymeric molecular weight, and types of surface modifications.^12^ Several features make chitosan and its derivatives promising carriers for gene delivery including biocompatibility, biodegradability, nontoxicity, antimicrobial activity, low immunogenicity, low cost, and easy accessibility.^9^, ^11^, ^12^, ^13^ The carboxymethylation of chitosan yields carboxymethyl chitosan (CMC) with superior solubility in physiological pH, increased antimicrobial and anti‐inflammatory activity, and easier cellular uptake.^14^, ^15^ In fact, CMC has been commercialized with a wide range of applications in biomedical fields, such as wound healing, biological imaging, tissue engineering, and drug delivery.^10^, ^12^, ^13^, ^16^ While CMC and other chitosan derivatives have been used as nanoparticle carriers for drug, DNA and siRNA delivery, the efficiency for chitosan‐mediated in vivo gene delivery remains relatively limited.

In this study, we exploited chitosan's biocompatibility, controlled release capability, and anti‐inflammatory activity, and sought to study whether a combination of CMC and Ad vectors offers an efficient gene therapy strategy. Our results showed that in the presence of optimal concentrations of CMC, Ad‐mediated transgene expression lasted up to 50 days after subcutaneous injection, and at least 7 days after intrahepatic injection. Histologic evaluation and immunohistochemical (IHC) analysis revealed that CMC effectively alleviated Ad‐induced host immune response. Using the CCl_4_‐induced experimental mouse model of chronic liver damage, we demonstrated that repeated intrahepatic administrations of Ad‐IL10 mixed with CMC effectively mitigated the development of hepatic fibrosis. Collectively, these results indicate that chitosan derivatives such as CMC can improve the prospect of using Ad vector for gene therapy by diminishing the host immune response while allowing readministration and sustained transgene expression.

## 2 MATERIALS AND METHODS

### 2.1 Cell culture and chemicals

HEK‐293 derivative 293pTP and RAPA cells were previously characterized and used for adenovirus packaging and subsequent amplification.^17^, ^18^ These cells were cultured in high glucose complete Dulbecco's modified Eagle's medium supplemented with 10% FBS (Cat# S711‐001S, Lonsera), 100 units of penicillin, and 100 μg of streptomycin at 37°C in 5% CO_2_ as described.^19^, ^20^, ^21^, ^22^, ^23^ CMC (cat# sc‐358091, CAS 83512‐85‐0) was purchased from Santa Cruz Biotechnology. CMC was dissolved in sterile PBS to prepare for a stock solution of 5% (wt/vol) and was kept at 4°C. Unless indicated otherwise, all other chemicals were purchased from Sigma‐Aldrich, Thermo Fisher Scientific, or Solarbio.

### 2.2 Construction, amplification, and purification of the recombinant adenoviruses Ad‐FLuc, Ad‐IL10, and Ad‐GFP

All recombinant adenoviruses were constructed by using the AdEasy technology.^24^ The Ad‐FLuc, which co‐expresses firefly luciferase (FLuc) and GFP, was generated as described in our previous studies.^25^, ^26^, ^27^ For the construction of Ad‐IL10, the coding region of human IL‐10 was PCR amplified and subcloned into an adenoviral shuttle vector, pAdTrack‐CMV. The resultant vector was used to generate the recombinant adenoviral plasmid pAd‐IL10 through homologous recombination with an adenoviral backbone vector in bacterial BJ5183 cells.^24^, ^28^, ^29^ The adenoviral plasmid pAd‐IL10 was subsequently linearized and used to generate recombinant adenovirus Ad‐IL10 in 293pTP or RAPA packaging cells. The Ad‐IL10 also co‐expresses GFP as a marker for tracking infection efficiency as described.^30^, ^31^ An analogous adenovirus expressing GFP only, Ad‐GFP, was used as a mock virus control.^32^, ^33^, ^34^

For direct in vivo injection studies, Ad‐FLuc, Ad‐IL10, and Ad‐GFP were amplified in large scale and purified through CsCl gradient ultracentrifugation, as described.^24^, ^35^, ^36^ The purified high titer adenovirus stocks were titered, aliquoted, and stored at −20°C. Desalting dialysis against sterile PBS was performed immediately prior to use.

### 2.3 Determination of the effect of CMC on Ad‐elicited acute and chronic host immune response

The use and care of animals in this study was approved by the Animal Care and Use Committee of The University of Chicago, Illinois, USA, and the Ethics Committee for Research and Experimental Animal Use of Chongqing Medical University, Chongqing, China. All experimental procedures followed the approved guidelines.

Subcutaneous or intrahepatic injection of Ad‐GFP mixed with or without CMC was carried out to assess Ad‐induced host immune response. For the acute response, two routes of Ad delivery were assessed. In the first route, C57BL/6J mice (*n* = 9, male, 6‐week old) were subcutaneously injected into both flanks with 10^10^ pfu (plaque forming unit) of Ad‐GFP mixed with either PBS alone or 1.3% CMC (wt/vol, in PBS) in 30 μl total volume. Three mice were sacrificed at 6, 24, and 72 h after injection. The injection sites were retrieved for histologic and IHC analyses. In the second route, C57BL/6J mice (*n* = 30, male, 6‐week old) were subjected to intrahepatic injection with 10^10^ pfu of Ad‐GFP mixed with either PBS alone (Ad‐GFP, control group) or 1.3% CMC (wt/vol, in PBS) (Ad‐GFP + CMC, treatment group) in 30 μl total volume. At 6, 24, and 72 h after injection, five mice from each group were sacrificed at each time point. Both serum and liver samples were retrieved for blood analysis and histologic evaluation, respectively.

For long‐term chronic immune response, C57BL/6J mice (*n* = 20, male, 6‐week old) were subjected to intrahepatic injection with 10^10^ pfu of Ad‐GFP mixed with either PBS alone (Ad‐GFP, control group) or 1.3% CMC (wt/vol, in PBS) (Ad‐GFP + CMC, treatment group) in 30 μl total volume. The intrahepatic injections were repeated once every 10 days. Five mice from each group were sacrificed at 4 and 8 weeks after the first injection. Both serum and liver samples were retrieved for blood analysis and histologic evaluation, respectively.

### 2.4 Subcutaneous and intrahepatic injection of Ad‐FLuc encapsulated with CMC

**FIGURE 1:**
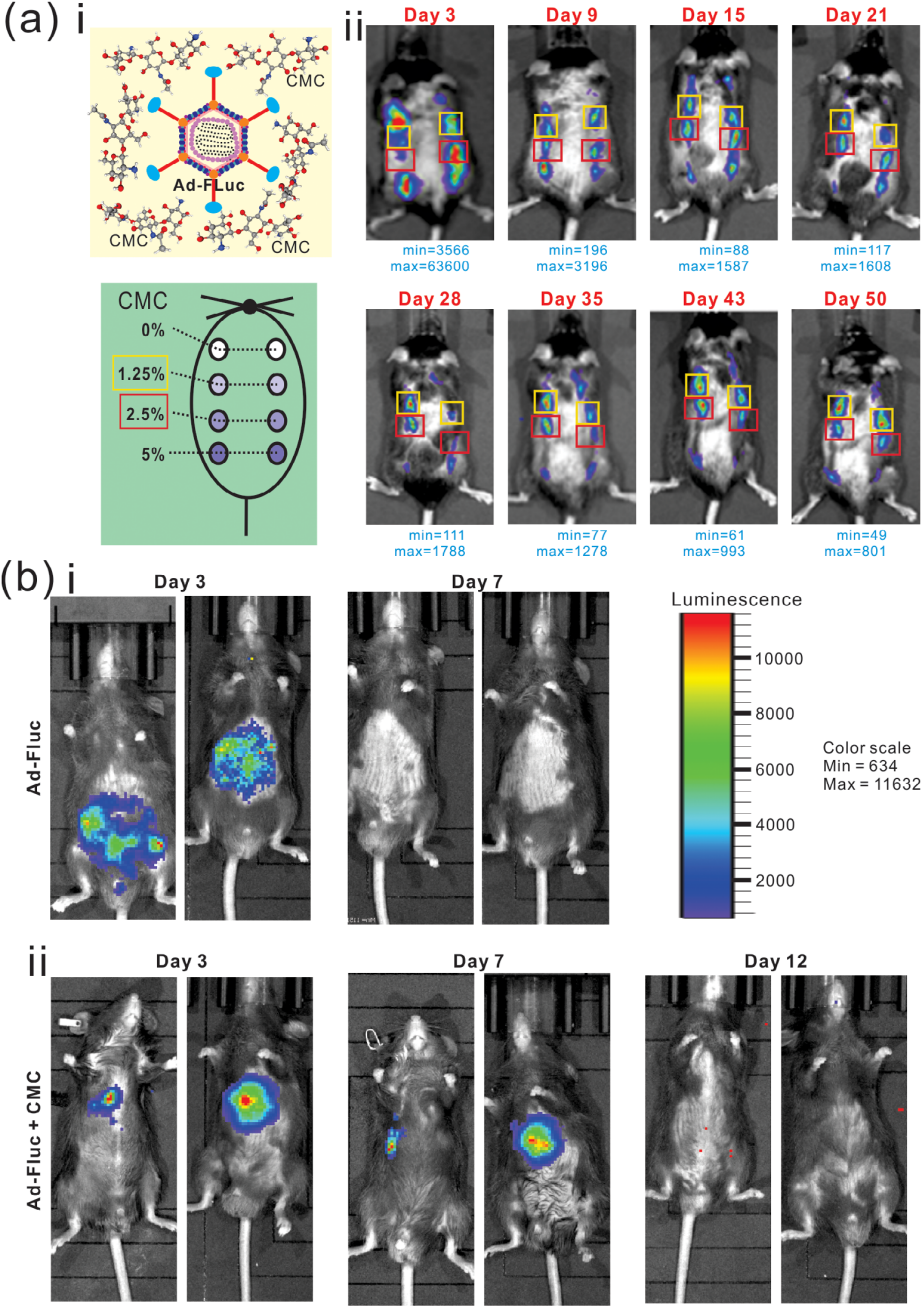
Optimal CMC concentrations prolong adenovirus‐mediated gene expression in vivo. (a) Optimal CMC concentrations for Ad‐FLuc expression after subcutaneous injection. The indicated concentrations (% in wt/vol) of CMC were prepared in 30 μl PBS, and mixed with 10^10^ infectious units of Ad‐FLuc adenovirus, followed by subcutaneous injections into the flanks of C57BL/6 mice (i). The animals were subjected to whole body Xenogen bioluminescence imaging at the indicated time points after injection (ii). The minimal and maximum signals are indicated for each time point. Representative images are shown. (b) Ad‐Fluc in 30 μl total volume of PBS (FLuc group) (i), or Ad‐FLuc with 1.3% (wt/vol, in PBS) CMC in 30 μl total volume of PBS (FLuc + CMC group) (ii), were intrahepatically injected and the mice were subjected to the whole‐body bioluminescence imaging at Days 3, 7, and 12, respectively. The normalized luminescence color scale is shown in the top right panel. Representative results are shown

Subcutaneous injection of the adenovirus Ad‐FLuc was used to determine the effect of various concentrations of CMC on adenovirus‐mediated transgene expression as described.^37^ Specifically, C57BL/6J mice (male, 6‐week old, *n* = 4) were subcutaneously injected into both flanks with 10^10^ pfu of Ad‐FLuc, which was premixed with 0% (wt/vol, in PBS), 1.25% (wt/vol, in PBS), 2.5% (wt/vol, in PBS), and 5% (wt/vol, in PBS) CMC (in 30 μl total volume) at each injection site, as shown in Figure 1a. Whole body optical bioluminescence imaging was performed at 3, 9, 15, 21, 28, 35, 43, and 50 days after injection by using the IVIS Spectrum In Vivo Imaging System (Perkin Elmer) with D‐Luciferin potassium (Gold Biotechnology, Inc.) as luciferase substrate as described.^38^, ^39^

For intrahepatic injection of Ad‐FLuc, C57BL/6J mice (male, 6‐week old, *n* = 4 each group) received intrahepatic injection of 10^10^ pfu of Ad‐FLuc, which was premixed with 0% CMC (wt/vol, in PBS), or 1.3% CMC (wt/vol, in PBS), all in 30 μl total volume, as shown in Figure S1. Whole body optical bioluminescence imaging was performed at 3, 7, and 12 days after injection by using the IVIS Spectrum in vivo imaging system as described.^40^, ^41^, ^42^

### 2.5 Establishment of the mouse model of chronic hepatic injury and fibrosis

The hepatic fibrosis model was established as described.^43^ Briefly, C57BL/6J mice (male, 6‐week old) were intraperitoneally injected with 2.0 μl/g body weight (g.b.w.) of 20% CCl_4_ solution (wt/vol, in olive oil) twice a week for up to 8 weeks as described.^43^ The control mice received intraperitoneal injections of 2.0 μl/g.b.w. olive oil twice a week. At each endpoint, serum levels of liver enzymes and liver histology were analyzed.

### 2.6 The effect of CMC‐encapsulated Ad‐IL10 on the mouse model of hepatic fibrosis

A total of 40 C57BL/6J mice (male, 6 weeks old) were randomly divided into four groups (10 mice each group). Ten mice were subjected to intraperitoneal injection of olive oil twice a week at 2.0 μl/g.b.w. and served as the control group. The other 30 mice were intraperitoneally injected twice a week with 2.0 μl/g 20% CCl_4_ solution (wt/vol, in olive oil) to establish experimental liver fibrosis. Concurrently with the first injection of CCl_4_ solution, 10 mice were intrahepatically injected with 10^10^ pfu of Ad‐GFP (in PBS, each mouse in 30 μl total volume; aka, Fibrosis model group), 10^10^ pfu of Ad‐IL10 (in PBS, each mouse in 30 μl total volume; aka, IL10 group), or 10^10^ pfu of Ad‐IL10 (in 1.3% CMC/PBS, each mouse in 30 μl total volume; aka, IL10 + CMC group). The Ad injections (mixed with or without CMC) were repeated once every 10 days. Animal body weight changes were recorded weekly. At weeks 4 and 8 after the first injection, five mice from each group were sacrificed. Both serum and liver samples were retrieved for blood analysis and histologic evaluation, respectively.

### 2.7 Determination of the serum levels of alanine aminotransferase (ALT), aspartate aminotransferase (AST), total bilirubin (TBIL), direct bilirubin (DBIL), and albumin (Alb)

Mouse cardiac blood collection was carried out as previously described.^37^, ^44^ Briefly, mice were first anesthetized by intraperitoneal injection of 3% sodium pentobarbital at 50 mg/kg body weight. An incision was made in upper‐middle abdomen across the abdominal and chest cavity to expose the liver and heart, and phlebotomized slowly from the left ventricle until reaching 500 μl for each mouse. The mice were then euthanized. The collected blood samples were centrifuged at 3500RPM for 10 min at RT. The upper portion of the serum samples was collected for assessing liver function parameters: ALT (Cat# C009‐2‐1, Nanjing Jiancheng Bioengineering Institute, China), AST (Cat# C010‐2‐1, Nanjing Jiancheng Bioengineering Institute), TBIL (Cat# C019‐1‐1, Nanjing Jiancheng Bioengineering Institute), and DBIL (Cat# C019‐2‐1, Nanjing Jiancheng Bioengineering Institute) and Alb (Cat# A028‐2‐1, Nanjing Jiancheng Bioengineering Institute).

### 2.8 Total RNA isolation and touchdown‐quantitative real‐time PCR (TqPCR) analysis

Total RNA from freshly‐prepared liver tissues was isolated by using the TRIZOL Reagent (Invitrogen) as described.^45^, ^46^ Total RNA was subjected to reverse transcription with hexamer and M‐MuLV reverse transcriptase (New England Biolabs). The cDNA products were further diluted and used as PCR templates. Gene‐specific qPCR primers were designed by using the Primer3 program (Table S1). TqPCR was carried out by using 2x SYBR Green qPCR Master Mix (Bimake) on a CFX‐Connect unit (Bio‐Rad Laboratories) as described.^47^ All TqPCR reactions were done in triplicate. *Gapdh* was used as a reference gene. Quantification of gene expression was carried out by using the 2^*−*ΔΔ*C*q^ method as described.^48^

### 2.9 Hematoxylin and eosin (H&E) analysis and Sirius red staining

The retrieved skin and liver samples were fixed with 4% paraformaldehyde and subjected to paraffin embedding, followed by sectioning. The slides were deparaffinized and used for H&E staining as previously described.^46^, ^49^, ^50^ The sections from the retrieved liver samples were deparaffinized and subjected to Sirius red staining (Picro Sirius Red solution, G1471, Solarbio) as previously reported.^43^

### 2.10 IHC staining

The sections from the above paraffin‐embedded skin and liver samples were also deparaffinized and subjected to IHC staining as described.^45^, ^48^, ^51^, ^52^ Specifically, the sections were deparaffinized and rehydrated. After antigen retrieval, the sections were subjected to immunostaining with antibodies against CD45 (1:100 dilution; Wanleibio; Cat# WL00922), CD54 (1:100 dilution; Wanleibio; Cat# WL02268), CD40L (1:50 dilution; Bimake; Cat. No. A5778), CD3D (1:50 dilution; Bimake; Cat. No. A5886), TNFα (1:200 dilution; Wanleibio; Cat# WL01896), IL1β (1:200 dilution; Wanleibio; Cat# WL00891), CD20 (1:200 dilution; Wanleibio; Cat# WL02883), IL10 (1:200 dilution; Wanleibio; Cat# WL03088), Collagen I (1:100 dilution; Wanleibio; Cat# WL0088), α‐SMA (1:50 dilution; Wanleibio; Cat# WL02510), or TIMP1 (1:100 dilution; Wanleibio; Cat# WL02342). The proteins of interest were detected with the biotin labeled goat anti‐rabbit IgG or anti‐mouse IgG/streptavidin‐HRP kit (SP Kit, SP‐9000, ZSGB‐Bio). Minus primary antibody and/or rabbit IgG and mouse IgG were used as negative controls. Staining results were recorded under a bright field microscope (Leica, DM4B).

### 2.11 Statistical analysis

Data were analyzed using GraphPad Prism 7 and presented as the mean ± standard deviations (SD). Statistical significance was determined by one‐way analysis of variance and the student's *t* test for the comparisons between groups. A value of *p* < 0.05 was considered statistically significant.

## 3 RESULTS

### 3.1 Optimal concentrations of CMC prolong adenovirus‐mediated transgene expression in vivo

We first tested the effect of CMC concentrations on Ad‐FLuc‐mediated in vivo luciferase expression. When 10^10^ pfu Ad‐FLuc was mixed with various concentrations of CMC at 0, 1.25, 2.5, and 5% (wt/vol in PBS) and subcutaneously injected into the flanks of immuno‐competent mice (Figure 1a, Panel i), whole body bioluminescence imaging analysis revealed that, while the FLuc signal in the 0% CMC group peaked at Day 3, but diminished at 15 days after injection, the injection sites in the 1.25% and 2.5% CMC groups exhibited detectable FLuc signal up to 50 days after injection (Figure 1a, Panel ii). Interestingly, the Ad‐FLuc mixed with 5% CMC did not exhibit higher signal activities than that with 1.25% or 2.5% CMC, indicating that high concentrations of CMC may hamper the release of adenoviral vectors and hence limit transgene expression (Figure 1a, Panel ii), which was further supported by the quantitative analysis of the bioluminescence imaging data (Figure S1A). Thus, these results suggest that the optimal CMC concentrations for sustained subcutaneous transgene expression may fall between 1.25% and 2.5%. We thus chose to use 1.3% CMC as the optimal concentration for all studies outlined below.

We also tested the duration of in vivo FLuc expression of intrahepatic injection of Ad‐FLuc mixed with or without 1.3% CMC. When the intrahepatic injection was carried out as illustrated in Figure S1B, we found that injections with or without 1.3% CMC exhibited strong FLuc signals at Day 3 (Figure 1b). However, the Ad‐FLuc+1.3% CMC group maintained relatively strong signals at Day 7 (although the signals were not readily detectable at Day 12), while the Ad‐FLuc alone group failed to display any FLuc activity under the same imaging condition (Figure 1b, Panel i vs. ii). Collectively, these results demonstrate that optimal concentrations of CMC (e.g., between 1.25% and 2.5%) can effectively prolong adenovirus‐mediated transgene expression in vivo.

### 3.2 CMC mitigates adenoviral vector‐elicited host immune and inflammatory responses in vivo

**FIGURE 2:**
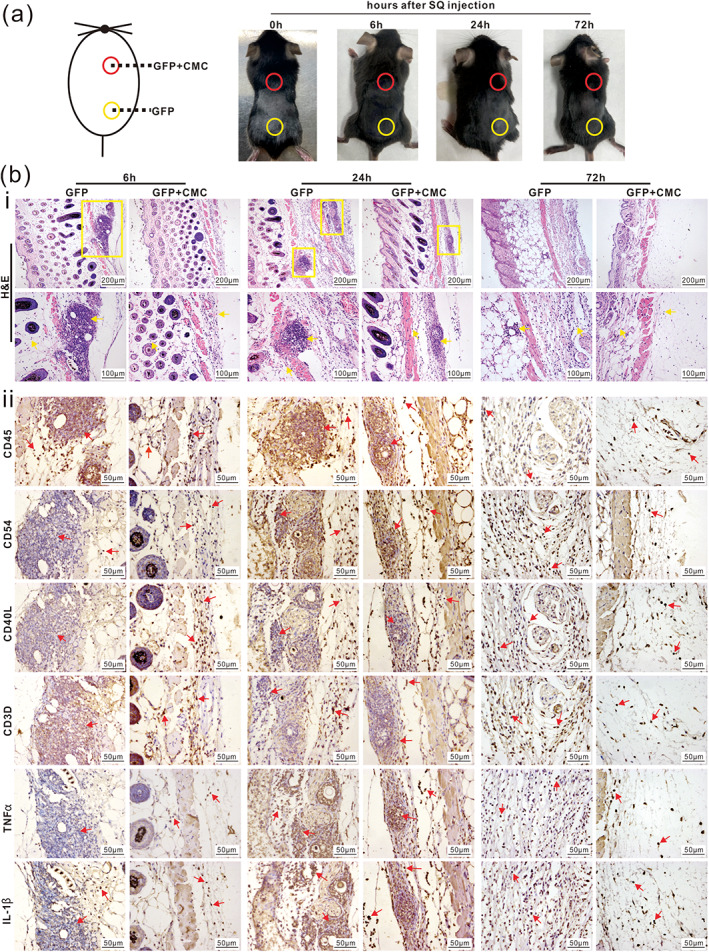
CMC effectively mitigates host acute immune response to subcutaneous adenovirus infection. (a) Ad‐GFP with (red cycle) or without (yellow cycle) 1.3% (wt/vol, in PBS) CMC in 30 μl total volume of PBS were injected subcutaneously into the back of C57BL/6J mice as shown. Mice were sacrificed at 6, 24, and 72 h after injection, respectively. The appearance of skin at injection site was documented. Representative images are shown. (b) Histologic evaluation and immunohistochemcial staining. The retrieved skin tissues from injection sites were subjected to H&E staining and inflammatory cells were indicated by yellow boxes (×100) and arrows (×200) (i). The retrieved tissues were further subjected to IHC staining assays with antibodies for CD45, CD54, CD40L, CD3D, TNFα, and IL1β (ii). Representative positively stained cells are indicated with red arrows (×400). Representative results are shown

We further analyzed the effect of CMC on adenovirus‐induced acute and chronic host immune response. When Ad‐GFP vector mixed with or without 1.3% CMC was subcutaneously injected into immuno‐competent mice, papules appeared at the injection sites of the Ad‐GFP group, but not the Ad‐GFP + CMC group at 24 h after injection (Figure 2a). H&E staining revealed that while a large number of inflammatory cells were presented in the whole skin layer, especially around the injection site under the dermis in the Ad‐GFP group at 6 and 24 h after injection, the Ad‐GFP + CMC group exhibited lower numbers of inflammatory cells in the skin, especially around the injection site at all three time points (Figure 2b, Panel i). Since mature dendritic cells (DCs) are important antigen presenting cells to activate T lymphocytes and secrete TNFα and IL1β to regulate the acute immune response,^53^, ^54^ we performed IHC to assess the presence of these inflammatory response cells, and found that the presence of CMC in Ad‐GFP subcutaneous injection effectively decreased the number of inflammatory cells (marked by CD45), mature DCs (marked by CD54), mature T lymphocytes (marked by CD40L and CD3D), as well as the expression of TNFα and IL1β, compared with that in the Ad‐GFP alone group (Figure 2b, Panel ii) and the negative staining controls (Figure S2A).

**FIGURE 3:**
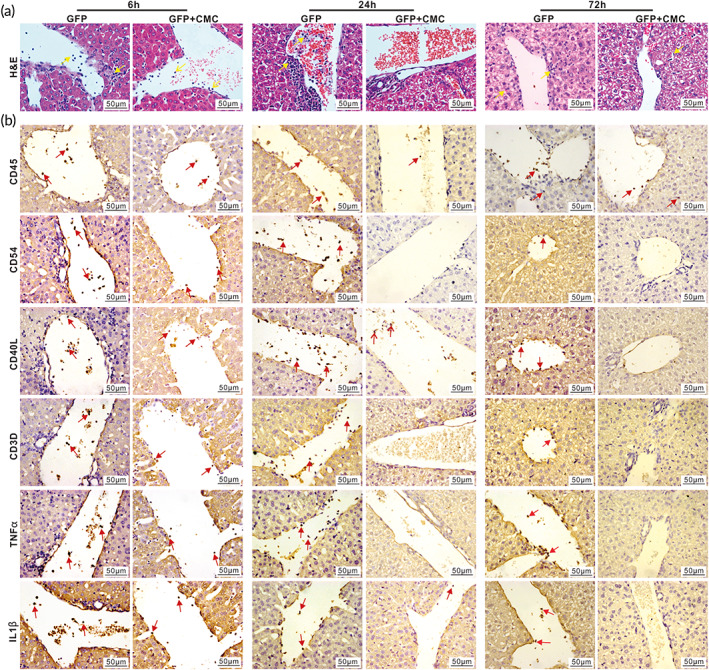
CMC diminishes host acute immune response of hepatic adenovirus infection. Ad‐GFP with (GFP + CMC group) or without (GFP group) 1.3% (wt/vol, in PBS) CMC in 30 μl total volume of PBS were intrahepatically injected into C57BL/6J mice. The mice were sacrificed at 6, 24, and 72 h after injection, respectively. The retrieved liver tissues were subjected to H&E staining. Representative inflammatory cells are indicated with yellow arrows (×400) (a). The retrieved liver tissues were further subjected to IHC staining with antibodies against CD45, CD54, CD40L, CD3D, TNFα, and IL1β (b). Representative positively stained cells are indicated with red arrows (×400). Representative results are shown

We also performed the intrahepatic injection of Ad‐GFP mixed with or without 1.3% CMC (wt/vol, in PBS) and analyzed both serum and liver samples retrieved at 6, 24, or 72 h after injection. Serum liver function parameter analysis indicated that the AST and ALT activities, along with TBIL, were higher in the Ad‐GFP alone group, compared with that in the Ad‐GFP + CMC group at 72 h after injection, although the DBIL level exhibited no significant differences in both groups (Figure S2B, Panels *a–d*). These serum results indicate that the addition of CMC mitigates adenovirus‐induced liver injury. While the gross appearance of the liver samples retrieved from the two group at 6, 24, and 72 h after injection did not show any apparent difference (Figure S2C), H&E analysis revealed that more inflammatory cells were seen in and around blood vessels in the Ad‐GFP group, compared with that in the Ad‐GFP + CMC group at 24 and 72 h after injection, respectively (Figure 3a). IHC staining analysis also confirmed that the addition of CMC reduced the number of inflammatory cells (marked by CD45), mature DCs (marked by CD54), mature T lymphocytes (marked by CD40L and CD3D), and down‐regulated the expression of TNFα and IL1β (Figures 3b and S2D). These results strongly suggest that CMC may effectively mitigate adenovirus‐induced host acute immune response in vivo.

### 3.3 CMC alleviates adenovirus‐induced chronic inflammatory injury of liver

**FIGURE 4:**
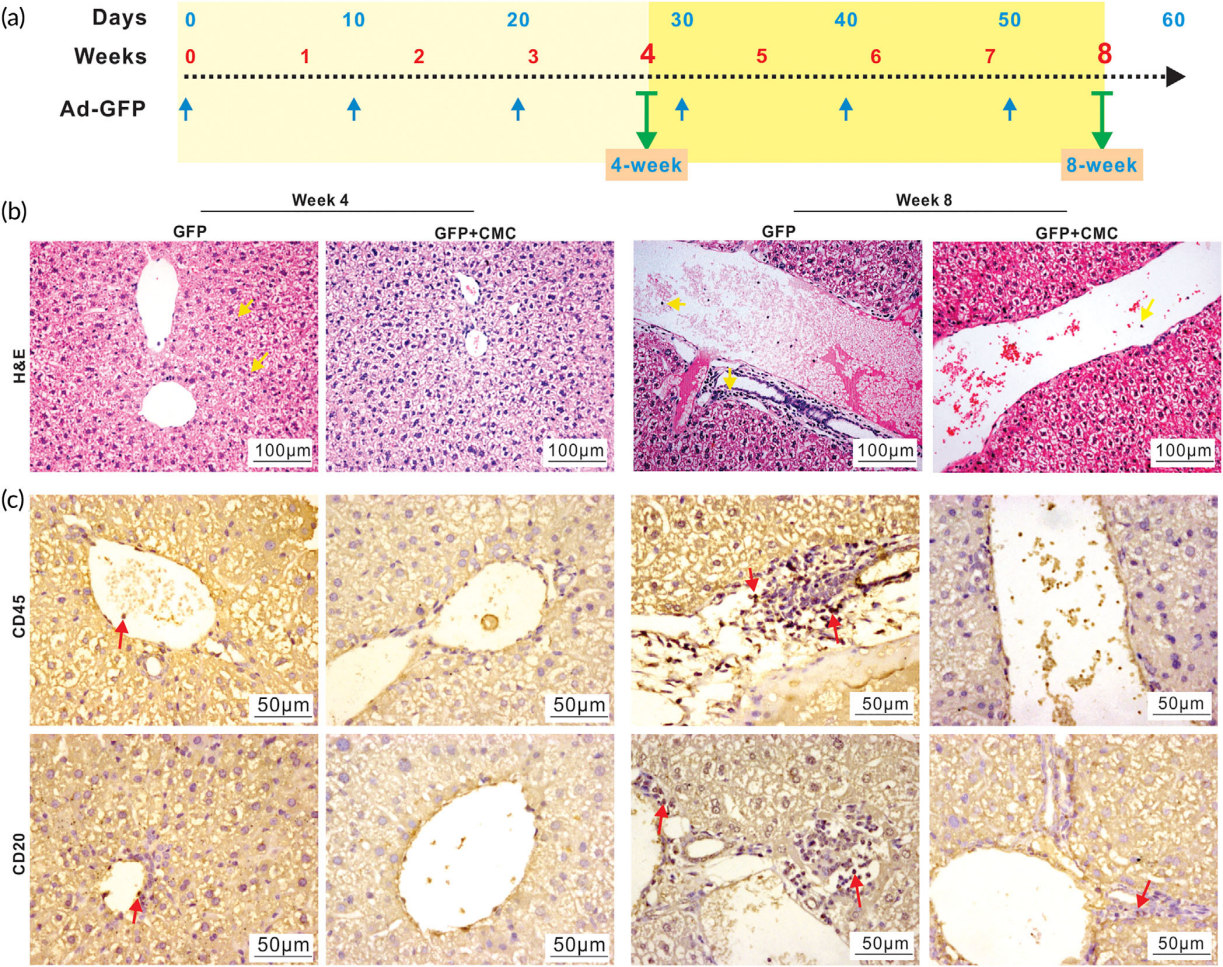
CMC alleviates chronic hepatic inflammation caused by repeated administrations of adenovirus vector in vivo. Ad‐GFP with or without 1.3% (wt/vol, in PBS) CMC in 30 μl total volume of PBS were intrahepatically injected once every 10 days; and the mice were sacrificed at Weeks 4 and 8, respectively (a). The retrieved liver tissues were subjected to H&E staining (b). Representative inflammatory cells are indicated with yellow arrows (×200). The retrieved liver tissues were also subjected to IHC staining with antibodies against CD45 and CD20 (c). Representative positively stained cells are indicated with red arrows (×400) (c). Representative results are shown

We next examined the effect of CMC on chronic inflammatory injury after repeated long‐term administration of adenovirus to the liver. Specifically, Ad‐GFP mixed with or without 1.3% CMC (wt/vol, in PBS) was intrahepatically injected once every 10 days for 4 and 8 weeks, respectively (Figure 4a). While the serum ALT and AST activities were elevated in the Ad‐GFP only group at Week 4, compared with that in the Ad‐GFP + CMC group (Figure S2E), no discernible differences in the gross appearance of the liver samples in both groups, compared with that of the normal control, were observed (Figure S2F). H&E staining revealed that the number of inflammatory cells around the central veins and portal areas in the Ad‐GFP + CMC group was lower than that in the Ad‐GFP only group at both Week 4 and Week 8 time points, respectively (Figure 4b).

Since chronic inflammatory injury usually activates both cellular immunity and B cell‐dependent humoral immunity,^54^ we performed IHC analysis and found that CMC markedly reduced the number of inflammatory cells (marked by CD45) and B cells (marked by CD20), compared with that in the Ad‐GFP only group (Figure 4c), while there were no obvious changes in the numbers of mature DCs (marked by CD54) and mature T lymphocytes (marked by CD3D and CD40L) (Figure S3A). However, CMC decreased the expression of TNFα at Week 4 and IL1β at Week 8, respectively (Figure S3A). Furthermore, the sirius red staining did not reveal any fibrous hyperplasia in portal area, and the cellular structure of hepatic lobule was relatively normal in both groups at Weeks 4 and 8 (Figure S3B). Collectively, the above results strongly suggest that CMC may provide a beneficial effect on alleviating chronic inflammatory injury after repeated administration of adenovirus to the liver.

### 3.4 Intrahepatic administration of CMC‐encapsulated Ad‐IL10 effectively alleviates hepatic fibrosis in a mouse model

While the exact mechanism remains to be fully understood, liver fibrosis is considered as a fibrotic healing response against a chronic injury or insult to the liver.^55^, ^56^ Here, we sought to establish an experimental hepatic fibrosis mouse model by intraperitoneal injection of carbon tetrachloride (CCl_4_). Briefly, C57BL/6J mice were intraperitoneally injected with 2.0 μl/g.b.w. of 20% CCl_4_ solution (in olive oil) twice a week for up to 8 weeks as described.^43^ The control mice received intraperitoneal injections of 2.0 μl/g.b.w. olive oil twice a week. Mouse body weight was monitored and exhibited no significant difference between the model group and control group (Figure S4A). At 4 and 8 weeks after injection, serum levels of liver enzymes were analyzed and found that ALT and AST (except Week 8) activities, but not TBIL, DBIL, and Alb (except for Week 4), were significantly elevated in the fibrosis model group (Figure S4B). Gross images of the retrieved liver samples revealed that fibrotic nodules were readily found in the model group at both Week 4 and Week 8, respectively, compared with that in the control group (Figure S4C, Panel *a*). H&E staining showed that inflammatory cells and balloon‐like changes of hepatocytes were presented in the liver tissues of the fibrosis model group, but not in the control group, at Weeks 4 and 8, respectively (Figure S4C, Panel *b*). Sirius red staining revealed that collagen was deposited around the central veins and portal areas of liver tissues in the fibrosis model group at Week 4; and more fibrous septa and pseudolobuli were readily observed in the fibrosis model group, but not in the control group, at Weeks 4 and 8, respectively (Figure S4C, Panel *c*). These results demonstrate that the mouse model of CCl_4_‐induced experimental hepatic fibrosis was successfully established.

**FIGURE 5:**
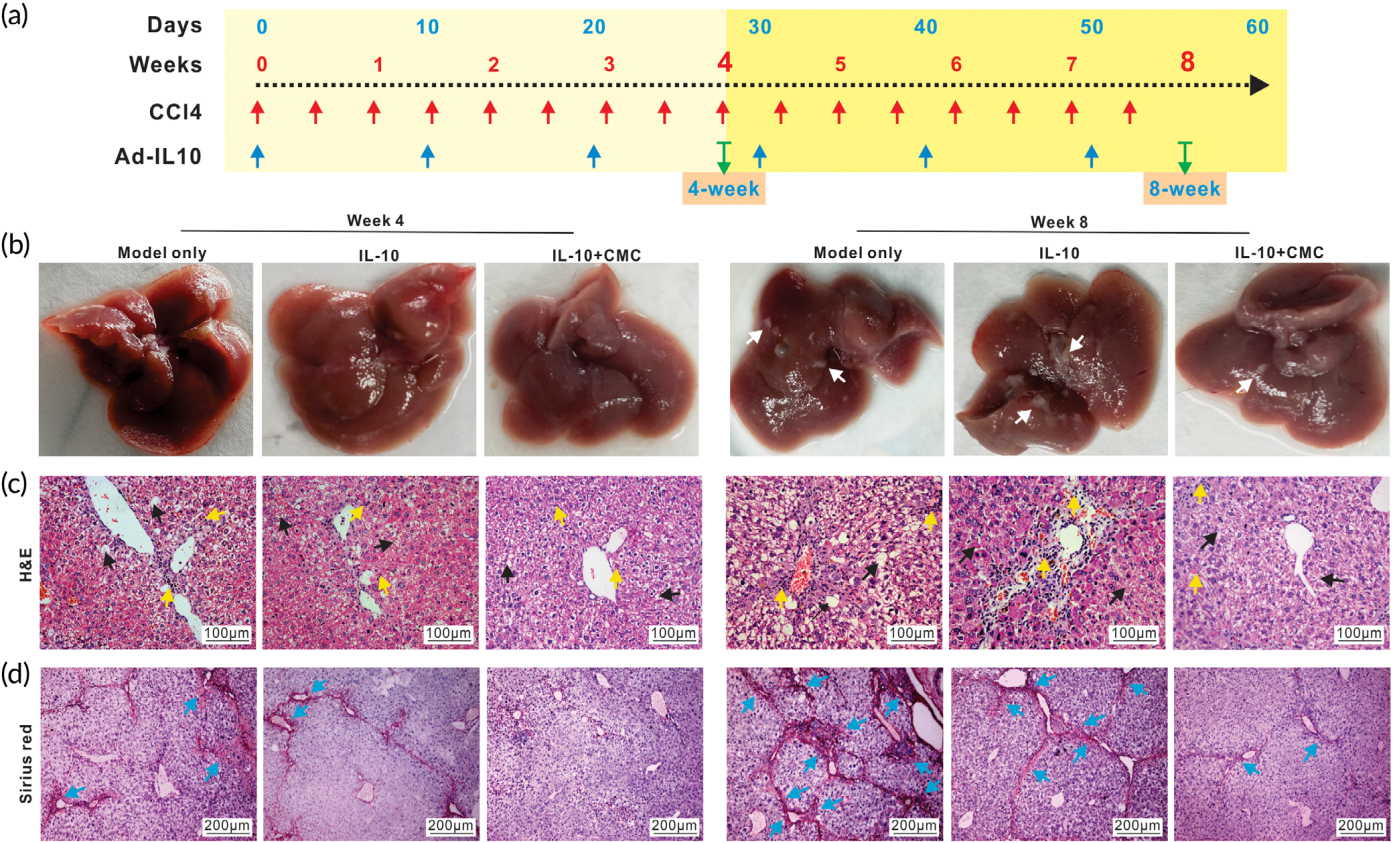
CMC‐encapsulated Ad‐IL10 mitigates the development and progression of chronic hepatic fibrosis in a mouse model. A mouse model of chronic hepatic fibrosis was induced by injecting CCl_4_ once every 3 days. Ad‐IL10 with (IL‐10 + CMC group) or without (IL‐10 group) 1.3% (wt/vol, in PBS) CMC in 30 μl total volume of PBS were intrahepatically injected once every 10 days into the CCl_4_ treated mice (a). The “model only” group received neither CMC and/or Ad‐IL10. The mice were sacrificed at Weeks 4 and 8, respectively. Macrophotographs of the gross appearance of the retrieved livers were obtained (b). Representative fibrotic nodules are indicated with white arrows (b). The retrieved liver tissues were subjected to H&E staining (c), and representative inflammatory cells are indicated by yellow arrows while representative hepatic fibrotic damage on hepatocytes and necrosis are indicated with black arrows (×200) (c). The retrieved liver tissues were further subjected to Sirius red staining (d), and the densely deposited collagens are indicated with blue arrows (×100) (d). Representative results are shown

Interleukin‐10 (IL10) is a cytokine produced by numerous activated immune cells such as B cells, mast cells, granulocytes, macrophages, DCs, and multiple T cell subsets, with plural and diverse cellular functions, and may play a beneficial preventive role in hepatic fibrosis.^55^, ^57^, ^58^, ^59^, ^60^, ^61^, ^62^ As a proof‐of‐concept experiment, we sought to investigate whether the intrahepatic administration of CMC‐encapsulated Ad‐IL10 would prevent and/or alleviate hepatic fibrosis in the mouse model of CCl_4_‐induced experimental hepatic fibrosis. As shown in Figure 5a, three groups were set up: the fibrosis model only, the Ad‐IL10 group, and Ad‐IL10 + CMC group, in which all mice were treated with CCl_4_ twice a week for 4 and 8 weeks, while the mice in the Ad‐IL10 and Ad‐IL10 + CMC groups also received intrahepatic injections of the Ad‐IL10 and Ad‐IL10 + CMC, respectively, once every 10 days for 4 and 8 weeks.

While the mouse body weight of the three groups did not show any significant difference (Figure S5A), the serum ALT of the IL‐10 + CMC group increased at Week 4, and the serum DBIL level of the IL‐10 group increased compared with that in the fibrosis group at Week 4 (Figure S5B). The gross images indicated that while the liver surface in all three groups was rough and granular at both Weeks 4 and 8, the number of white nodules on the liver surface in the Ad‐IL10 group and the Ad‐IL10 + CMC group was less than that in the fibrosis model group at Week 8 (Figure 5b). H&E staining showed that many inflammatory cells and the balloon‐like hepatocytes could be observed in the liver tissues retrieved from the fibrosis model group, to a lesser extent in the Ad‐IL10 group, compared with that in the Ad‐IL10 + CMC group (Figure 5c). Sirius red staining further confirmed that more collagen deposition and the formation of fibrous septa and pseudolobuli were readily found around the central veins and portal areas of liver tissues in the fibrosis model group, although much less in the Ad‐IL10 group at both Weeks 4 and 8 (Figure 5d). Most notably, the number of pseudolobuli and fibrous septa was drastically reduced in the liver tissues retrieved from the Ad‐IL10 + CMC group at both Weeks 4 and 8 (Figure 5d). These results demonstrate that adenovirus‐mediated delivery of IL10, especially when Ad‐IL10 was encapsulated with CMC, could effectively alleviate CCl_4_‐induced hepatic fibrosis.

**FIGURE 6:**
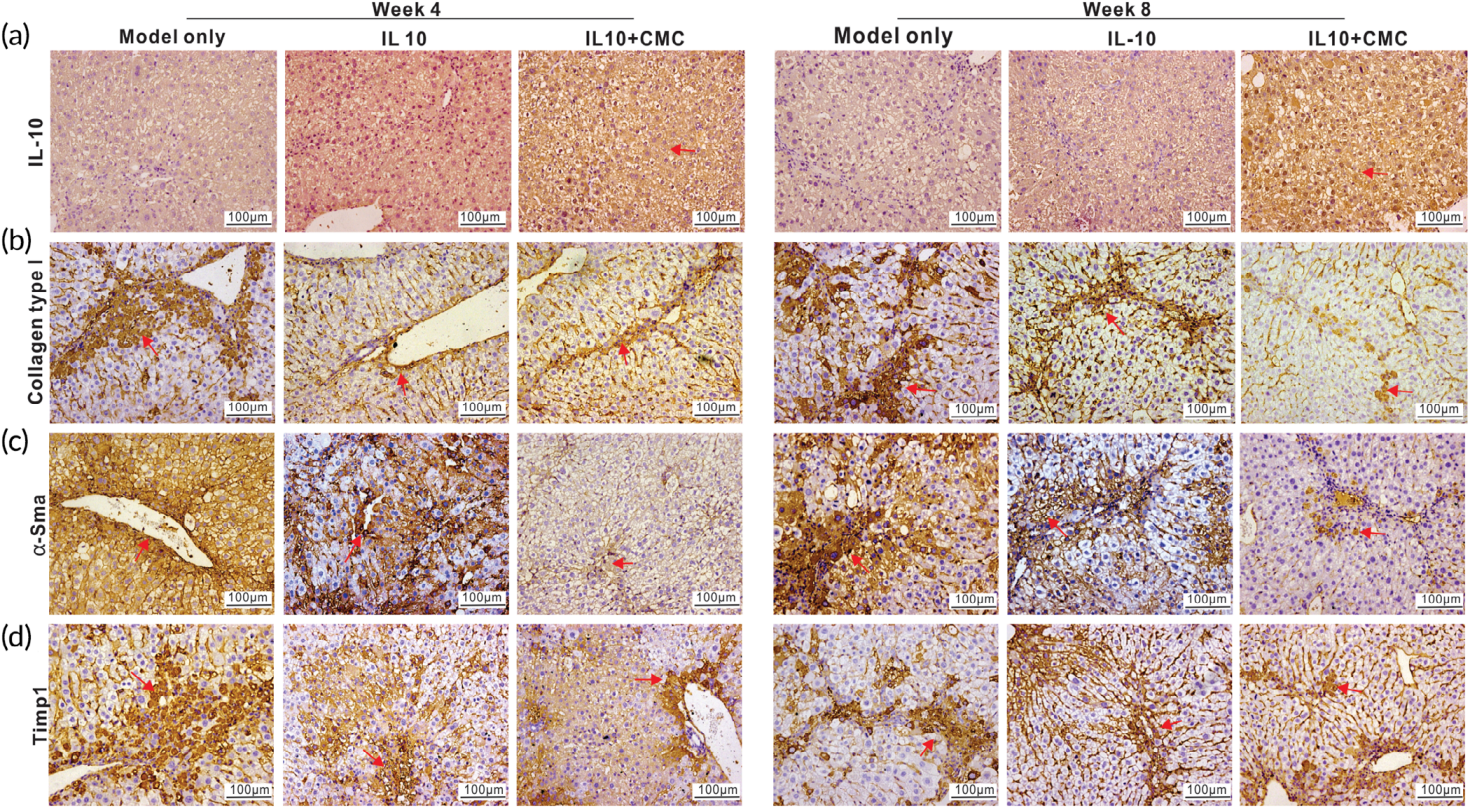
CMC‐encapsulated Ad‐IL10 diminishes the expression of fibrosis markers in CCl_4_‐induced mouse model of hepatic fibrosis. The retrieved liver samples from Week 4 and Week 8, as shown in Figure 5, were subjected to IHC staining with antibodies against IL‐10 (a) and fibrosis marker genes Collagen I (b), α‐Sma (c), and Timp1 (d). Representative positive stains are indicated with red arrows (×200). Representative results are shown

It has been well established that hepatic staller cells (HSCs) are the main myofibroblast progenitor cells and key effectors of fibrogenic response.^56^ During liver injury, activated HSCs progressively lose their star‐shaped morphology and their lipid droplets, and produce abundant extracellular matrix components such as Type I, III, and IV collagens, fibronectin, laminin, and proteoglycans, and pro‐inflammatory mediators, as well as expressing high levels of alpha smooth muscle actin (α‐Sma) and tissue inhibitor of metalloproteinase 1 (Timp1) that contribute to the change from adipocytic phenotype to profibrogenic and inflammatory phenotype. The qPCR analysis indicated that while the expression of IL‐10 increased, the expression of Type I collagen, α‐Sma, and Timp1 significantly decreased both in the Ad‐IL10 group and the Ad‐IL10 + CMC group, compared with that in the fibrosis model group, although the decrease in the Ad‐IL10 + CMC group was more pronounced than that in the Ad‐IL10 group (Figure S5C). Furthermore, the IHC staining analysis revealed the high expression of IL‐10 in the Ad‐IL10 + CMC group, compared with that in other two groups (Figure 6a). As expected, the expression of collagen I, α‐Sma, and Timp1 was most profoundly down‐regulated in the Ad‐IL10 + CMC group, compared with that in the fibrosis model group and the Ad‐IL10 group (Figures 6b–d and S5D). Collectively, the above results strongly suggest that CMC‐encapsulated Ad‐IL10 may effectively control the release and production of IL‐10 through intrahepatic injection and subsequently alleviate CCl_4_‐induced hepatic fibrosis in a mouse model.

## 4 DISCUSSION

Viral vector‐mediated gene delivery remains one of the most preferred approaches for liver‐directed gene therapy.^1^, ^2^, ^3^ Among the viral vectors, although AAV‐based gene therapy has recently received a great deal of attention due to AAV's favorable biosafety profile and reduced host immune response,^1^, ^4^, ^5^, ^7^, ^8^ Ad vector remains as a vector of choice in many cases including liver‐directed gene therapy, because it has at least two advantages over AAV and other viral vectors: it has high gene transfer efficiency, and it is easy to scale up for mass production with high titer.^6^, ^7^ However, two major shortcomings for Ad vector as a gene delivery system are the relatively short‐term of transgene expression and the solicitation of host immune response. Thus, overcoming these limitations would improve the prospect of using Ad vector as a preferred gene delivery vehicle for gene therapy including liver‐directed gene therapy.

In this study, we demonstrated that CMC could effectively prolong Ad‐mediated transgene gene expression in vivo, while reducing Ad‐induced host immune response, leading to sustained transgene expression. Using whole body optical imaging analysis, we showed that, in the presence of optimal concentrations (e.g., 1.25%–2.5% wt/vol) of CMC, Ad‐mediated firefly luciferase (FLuc) expression lasted up to 50 days after a single subcutaneous injection, and at least 7 days after intrahepatic injection. Histologic evaluation and IHC analysis revealed that CMC effectively alleviated Ad‐induced host immune response. Interestingly, we found that CMC at higher concentrations (e.g., 5% wt/vol) seemingly decreased FLuc expression, suggesting that high concentrations of CMC may prevent the timely release of Ad vectors and thus limit Ad‐mediated transgene delivery efficiency. While the exact mechanism through which CMC prolongs Ad‐mediated transgene expression in vivo remains to be fully understood, it is possible that the complex polymer structure of CMC controls the sustained release of Ad vector, and that the alleviation of Ad‐induced host immune response may further contribute to the sustained transgene expression in vivo.

Due to the transient expression nature of Ad‐mediated gene delivery, re‐administration is a desirable strategy to achieve sustained transgene expression in vivo. However, repeated injections of Ad vector will not only solicit strong host immune response, but also produce neutralizing antibodies to rapidly eliminate Ad vector in vivo. It is conceivable that CMC's ability to alleviate host immune response may provide an opportunity for the repeated administrations of Ad vector in vivo. To provide a proof‐of‐concept study, we established a CCl_4_‐induced experimental mouse model of chronic liver damage, and demonstrated that repeated intrahepatic injections, once every 10 days for up to six times, of Ad‐IL10 mixed with CMC effectively alleviated the development of hepatic fibrosis. It has been reported that IL10 exhibits anti‐hepatic fibrosis activity.^55^, ^57^, ^58^, ^59^, ^60^, ^61^, ^62^ Since the pathogenic process of hepatic fibrosis is highly complex, it is conceivable that other contributing factors may be explored and targeted through Ad‐mediated gene therapy in combination with CMC. Collectively, our results strongly indicate that chitosan derivatives such as CMC provide apparent benefit for Ad‐mediated in vivo gene delivery by diminishing host immune response while allowing sustained transgene expression. Nonetheless, it remains to be investigated whether CMC can prolong the transgene expression of non‐secreted proteins mediated by adenoviral vectors in vivo.

In this study, we chose to use CMC as the Ad vector delivery vehicle because CMC is widely available and has been approved for several preclinical and clinical applications such as wound healing dressing, biological imaging, tissue engineering, and controlled drug release.^10^, ^12^, ^13^, ^16^ It is conceivable that other chitosan derivatives may also be used as Ad vector delivery carriers.^9^, ^11^, ^12^, ^13^ The chemical versatility of chitosan and its derivatives is reflected by its ability to form a poly‐cationic charged polymer at physiological pH, and by its modifiable molecular weight and types of surface modifications, which in turn impact chitosan's chemical and biological properties.^12^ In fact, numerous studies have been devoted to modify and optimize CMC and/or other chitosan derivatives.^10^, ^11^, ^12^, ^15^ Early studies reported that both linear and branched polyethylenimine (PEI)‐graft‐chitosan copolymers functioned as efficient DNA/siRNA delivery vehicles both in vitro and in vivo.^63^, ^64^ Liang et al.^65^ reported that tetradecyl‐quaternized CMC/lipid cation polymeric liposomes had low cytotoxicity and could be used for cancer cell‐targeted gene delivery although the in vivo gene delivery efficacy of these copolymers has not been extensively investigated. Lee et al.^66^ demonstrated that a novel hybrid polymer, glycol chitosan–methyl acrylate‐low molecular weight PEI (GMP), spontaneously assembled with plasmid DNA into nanorods and functioned as a non‐viral vector for gene delivery in vitro. It was also shown that the chitosan and poly(ethylene glycol)‐grafted (PEGylated) chitosan nanoparticles, encapsulating β‐catenin siRNA, effectively decreased β‐catenin protein levels in colon cancer cells in vitro.^67^ Interestingly, a CMC‐modified polyamidoamine dendrimer achieved pH‐sensitive drug release in response to tumor microenvironment pH changes.^68^ More recently, it has been reported that a reactive oxygen species‐sensitive hydrogel with strong free radical scavenging ability was prepared by introducing the thione group into CMC hydrogel, which enhanced CMC's wound healing efficacy.^69^ Thus, some of these modified CMC and chitosan derivatives may be further explored as potential Ad delivery carriers.

It is worth noting that many polymers such as PEI, poly(ethylene glycol), poly(l‐lysine) (PLL), polyamidoamine dendrimer (PAMAM), and poly (aminoethers) have been exploited to enhance Ad vector‐mediated gene delivery, mostly by evading host immune response and/or redirecting tropism.^70^, ^71^, ^72^ However, our results demonstrate that CMC and potentially other chitosan derivatives may be a superior choice of polymeric helper for Ad gene therapy due to their exceptional properties, including biocompatibility, biodegradability, non‐cytotoxicity, antimicrobial and anti‐inflammation activity, low immunogenicity, inexpensiveness, and accessibility.

## 5 CONCLUSIONS

We sought to overcome the transient expression nature and strong host immune response associated with Ad‐mediated gene therapy by exploiting CMC's biocompatibility, controlled release capability and anti‐inflammatory activity. Our results demonstrated that in the presence of optimal concentrations of CMC, Ad‐mediated transgene expression lasted up to 50 days after subcutaneous injection, and at least 7 days after intrahepatic injection, respectively. Histologic evaluation and IHC analysis revealed that CMC effectively alleviated Ad‐induced host immune response. In our proof‐of‐principle study using the CCl_4_‐induced experimental mouse model of chronic liver damage, we showed that repeated intrahepatic administrations of Ad‐IL10 mixed with CMC effectively alleviated the development of hepatic fibrosis. Collectively, these results indicate that chitosan derivatives such as CMC can provide a beneficial effect for Ad‐mediated in vivo gene delivery by diminishing the host immune response while allowing sustained transgene expression.

## AUTHOR CONTRIBUTIONS

**Yannian Gou:** Conceptualization (equal); data curation (equal); formal analysis (equal); methodology (equal); validation (equal); writing – original draft (equal); writing – review and editing (equal). **Yaguang Weng:** Conceptualization (equal); formal analysis (equal); project administration (equal); resources (equal); supervision (supporting); writing – original draft (supporting); writing – review and editing (equal). **Qian Chen:** Data curation (equal); formal analysis (equal); methodology (equal); project administration (equal); writing – original draft (supporting); writing – review and editing (equal). **Jinghong Wu:** Data curation (equal); formal analysis (equal); methodology (equal); writing – review and editing (equal). **Hao Wang:** Data curation (equal); formal analysis (equal); methodology (equal); resources (equal); validation (equal); writing – review and editing (equal). **Jiamin Zhong:** Data curation (equal); formal analysis (equal); methodology (equal); resources (equal); writing – review and editing (equal). **Yang Bi:** Formal analysis (equal); methodology (equal); resources (equal); validation (equal); writing – review and editing (equal). **Daigui Cao:** Conceptualization (supporting); data curation (equal); methodology (equal); project administration (equal); writing – review and editing (equal). **Piao Zhao:** Data curation (equal); formal analysis (equal); methodology (equal); resources (equal); writing – review and editing (equal). **Xiangyu Dong:** Formal analysis (equal); methodology (equal); resources (equal); writing – review and editing (equal). **Meichun Guo:** Formal analysis (equal); methodology (equal); resources (equal); writing – review and editing (equal). **William Wagstaff:** Formal analysis (equal); methodology (equal); resources (equal); writing – original draft (supporting); writing – review and editing (equal). **Bryce Hendren‐Santiago:** Formal analysis (equal); methodology (equal); resources (equal); writing – original draft (supporting); writing – review and editing (equal). **Connie Chen:** Formal analysis (equal); methodology (equal); resources (equal); writing – original draft (supporting); writing – review and editing (equal). **Andrew Youssef:** Formal analysis (equal); methodology (equal); resources (equal); writing – original draft (supporting); writing – review and editing (equal). **Rex C. Haydon:** Conceptualization (equal); formal analysis (equal); project administration (equal); supervision (supporting); writing – original draft (supporting); writing – review and editing (equal). **Hue H. Luu:** Conceptualization (supporting); formal analysis (equal); project administration (equal); supervision (supporting); writing – original draft (supporting); writing – review and editing (equal). **Russell R. Reid:** Conceptualization (supporting); formal analysis (equal); funding acquisition (supporting); supervision (supporting); writing – original draft (equal); writing – review and editing (equal). **Le Shen:** Conceptualization (equal); formal analysis (equal); funding acquisition (supporting); supervision (equal); writing – original draft (supporting); writing – review and editing (equal). **Tong‐Chuan He:** Conceptualization (lead); data curation (equal); formal analysis (equal); funding acquisition (equal); project administration (equal); supervision (lead); validation (equal); writing – original draft (lead); writing – review and editing (equal). **Jiaming Fan:** Conceptualization (equal); formal analysis (equal); funding acquisition (lead); methodology (equal); supervision (lead); writing – original draft (lead); writing – review and editing (lead).

## CONFLICTS OF INTEREST

The authors declare no conflicts of interest.

### PEER REVIEW

The peer review history for this article is available at https://publons.com/publon/10.1002/btm2.10306.

## Supporting information


**Table S1** List of TqPCR Primers
**Figure S1**: Location for intrahepatic adenovirus injection into mice
**Figure S2**: IHC staining controls, serum levels of liver function parameters, and representative gross liver images
**Figure S3**: IHC staining results for liver samples injected with Ad mixed with or without CMC
**Figure S4**: Characterization of the CCl4‐induced liver fibrosis mouse model
**Figure S5**: The effect of Ad‐IL10 with or without CMC on CCl4‐induced liver fibrosis

## Data Availability

The authors confirm that the data supporting the findings of this study are available within the article and its supplementary materials, or available from the corresponding authors upon reasonable request.
