# Supplementary material for: Carboxymethyl chitosan prolongs adenovirus‐mediated expression of IL‐10 and ameliorates hepatic fibrosis in a mouse model
Source: Bioeng Transl Med. 2022 Mar 10;7(3):e10306. doi: 10.1002/btm2.10306 (PMC9472002; doi:10.1002/btm2.10306)
Supplement: Supplementary file 1 — Table S1 List of TqPCR Primers Figure S1: Location for intrahepatic adenovirus injection into mice Figure S2: IHC staining controls, serum levels of liver function parameters, and representative gross liver images Figure S3: IHC staining results for liver samples injected with Ad mixed with or without CMC Figure S4: Characterization of the CCl4‐induced liver fibrosis mouse model Figure S5: The effect of Ad‐IL10 with or without CMC on CCl4‐induced liver fibrosis [file BTM2-7-e10306-s001.pdf]

Carboxymethyl chitosan (CMC) prolongs adenovirus-mediated expression of IL-10 and ameliorates hepatic fibrosis in a mouse model

Running Title: CMC encapsulated Ad-IL10 mitigates mouse hepatic fibrosis

Yannian Gou[1,2], Yaguang Weng[1], Qian Chen[3], Jinghong Wu[1], Hao Wang[1,2], Jiamin Zhong[1,2], Yang Bi[2,4], Daigui Cao[2,5], Piao Zhao[1,2,6], Xiangyu Dong[1], Meichun Guo[1], William Wagstaff[2], Bryce Hendren-Santiago[2], Connie Chen[2], Andrew Youssef[2], Rex C. Haydon[2], Hue H. Luu[2], Russell R. Reid[2,7], Le Shen[2,8], Tong-Chuan He[2,7,8]* and Jiaming Fan[1,2]*

1. Ministry of Education Key Laboratory of Diagnostic Medicine, and Department of Clinical Biochemistry, School of Laboratory Medicine, Chongqing Medical University, Chongqing 400016, China
2. Molecular Oncology Laboratory, Department of Orthopaedic Surgery and Rehabilitation Medicine, The University of Chicago Medical Center, Chicago, IL 60637, USA
3. Health Management Center, Deyang People's Hospital, Deyang, Sichuan 618000, China
4. Stem Cell Biology and Therapy Laboratory of the Pediatric Research Institute, the National Clinical Research Center for Child Health and Disorders, and Ministry of Education Key Laboratory of Child Development and Disorders, the Children's Hospital of Chongqing Medical University, Chongqing 400016, China
5. Department of Orthopaedic Surgery, the Affiliated Hospital of the University of Chinese Academy of Sciences, and Chongqing General Hospital, Chongqing 400021, China
6. Department of Orthopaedic Surgery, the First Affiliated Hospital of Chongqing Medical University, Chongqing 400016, China
7. Craniofacial Suture Biology Laboratory, Department of Surgery Section of Plastic Surgery, The University of Chicago Medical Center, Chicago, IL 60637, USA
8. Department of Surgery, The University of Chicago Medical Center, Chicago, IL 60637, USA

* Corresponding authors.

CORRESPONDENCES

T.-C. He, MD, PhD
Molecular Oncology Laboratory
The University of Chicago Medical Center
Chicago, IL 60637, USA
Tel. (773) 702-7169
Fax: (773) 834-4598
Email: tche@uchicago.edu

Jiaming Fan, MD, PhD
Ministry of Education Key Laboratory of Diagnostic Medicine
Department of Clinical Biochemistry
School of Laboratory Medicine
Chongqing Medical University
Chongqing, 400016, China
Tel. 011-86-23-6848 5240
Email: fanjiaming1988@cqmu.edu.cn

Supporting Materials

Table S1. List of TqPCR Primers

Table S1: List of TqPCR Primers			
Gene	Forward primer	Reverse primer	Accession No.
Mouse Gapdh	ATGCCATCACTGCCACCC	GCCAGTGAGCTTCCCGTT	NM_001289726.1
Mouse IL-10	GCAGAGAAGCATGGCCCA	TGCTCCACTGCCTTGCTC	NM_010548.2
Mouse Collagen type I	GAACAGCTGGCCTCCCTG	GGCCAGGAGCTCCGTTTT	NM_007742.4
Mouse α-Sma	CAGGCTGTGCTGTCCCTC	CAGCCAAGTCCAGACGCA	NM_007392.3
Mouse Timp1	TTGGGGCATGTGCAGAGG	CGCACAGCCTGGTTACCA	NM_001044384.1

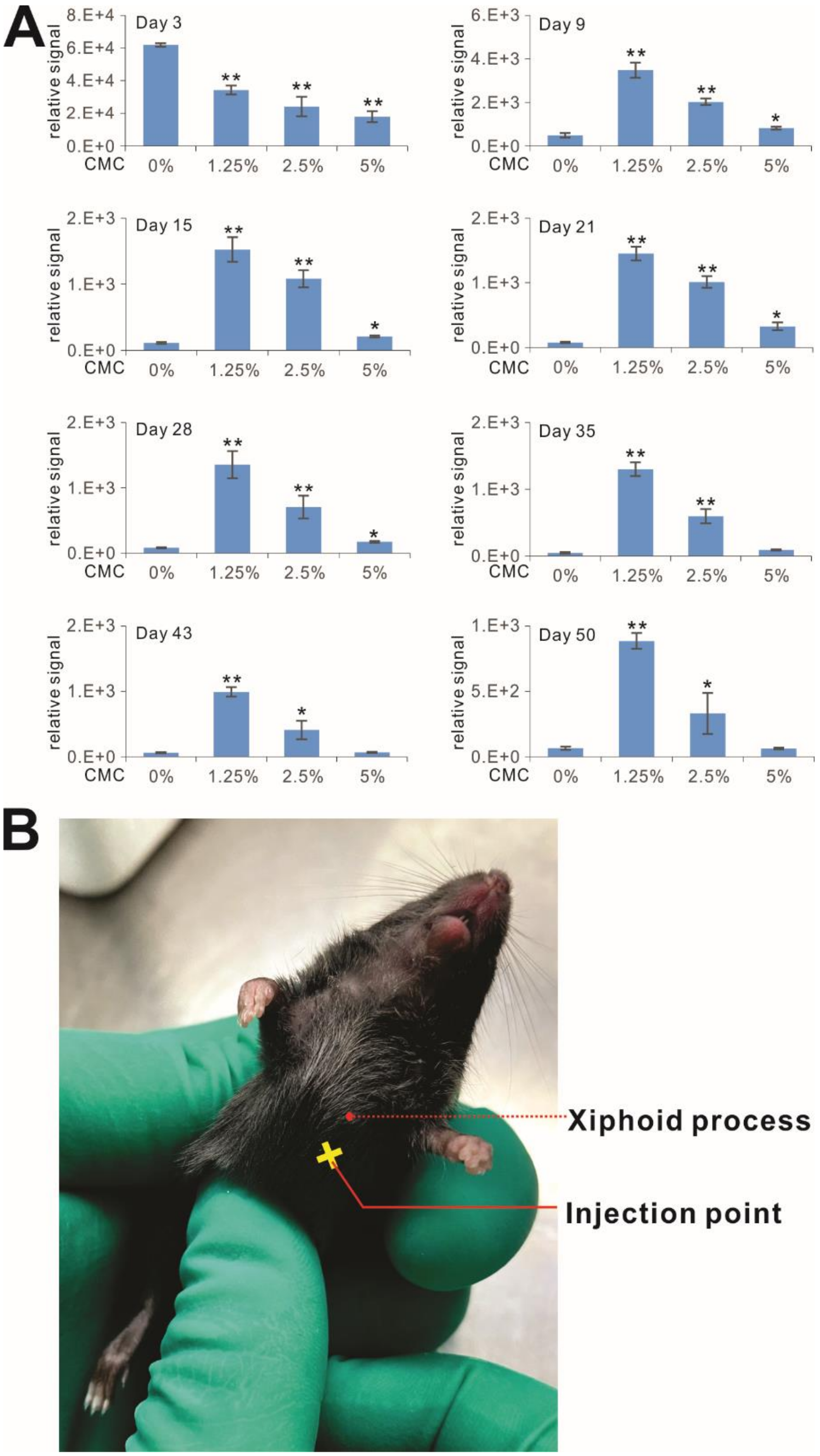

Figure S1. (A) Quantitative analysis of the Xenogen bioluminescence imaging data. The average relative signals (in photons/sec/cm2/steradian) were calculated for each time point by using Xenogen Living Image software. "*" $p<0.05$, "**" $p<0.01$, compared with that of the 0% CMC group. **(B) Location for intrahepatic adenovirus injection into mice**. The mouse xiphoid process served as a reference point. A 25G needle was inserted 5mm vertically in depth at 5~8mm below the xiphoid process reference point.

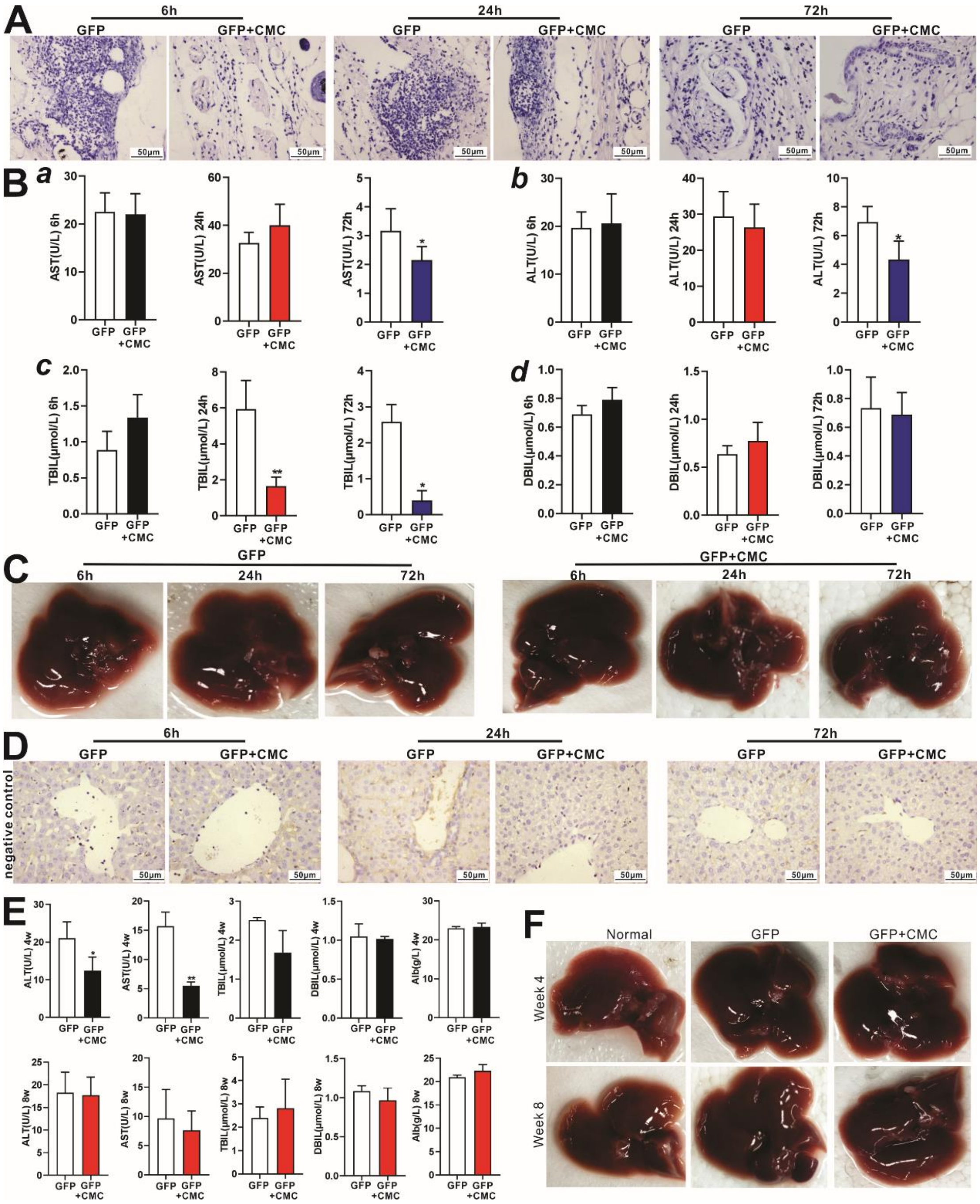

Figure S2. (A) IHC negative controls for **Fig. 2B-b**. The paraffin sections of skin samples at 6h, 24h and 72h were subjected to IHC staining without any primary antibody (x400). **(B)** The levels of serum AST (***a***), ALT (***b***), TBDL (***c***) and DBIL (***d***) at 6h, 24h and 72h for the samples in the **Fig. 3** were measured. "**" p < 0.01, "*" p < 0.05, GFP group vs. GFP+CMC group. **(C)** Representative gross images of liver samples in **Fig. 3** were obtained at 6h, 24h and 72h, respectively. **(D)** Paraffin sections of liver samples in **Fig. 3** were subjected to IHC staining without any primary antibody (x400). **(E)** The levels of serum ALT, AST, TBDL, DBIL and Alb in **Fig. 4** were measured at week 4 and 8, respectively. "**" p < 0.01, "*" p < 0.05, GFP group vs. GFP+CMC group. **(F)** Representative gross images of liver samples in **Fig. 4** were obtained at weeks 4 and 8, respectively.

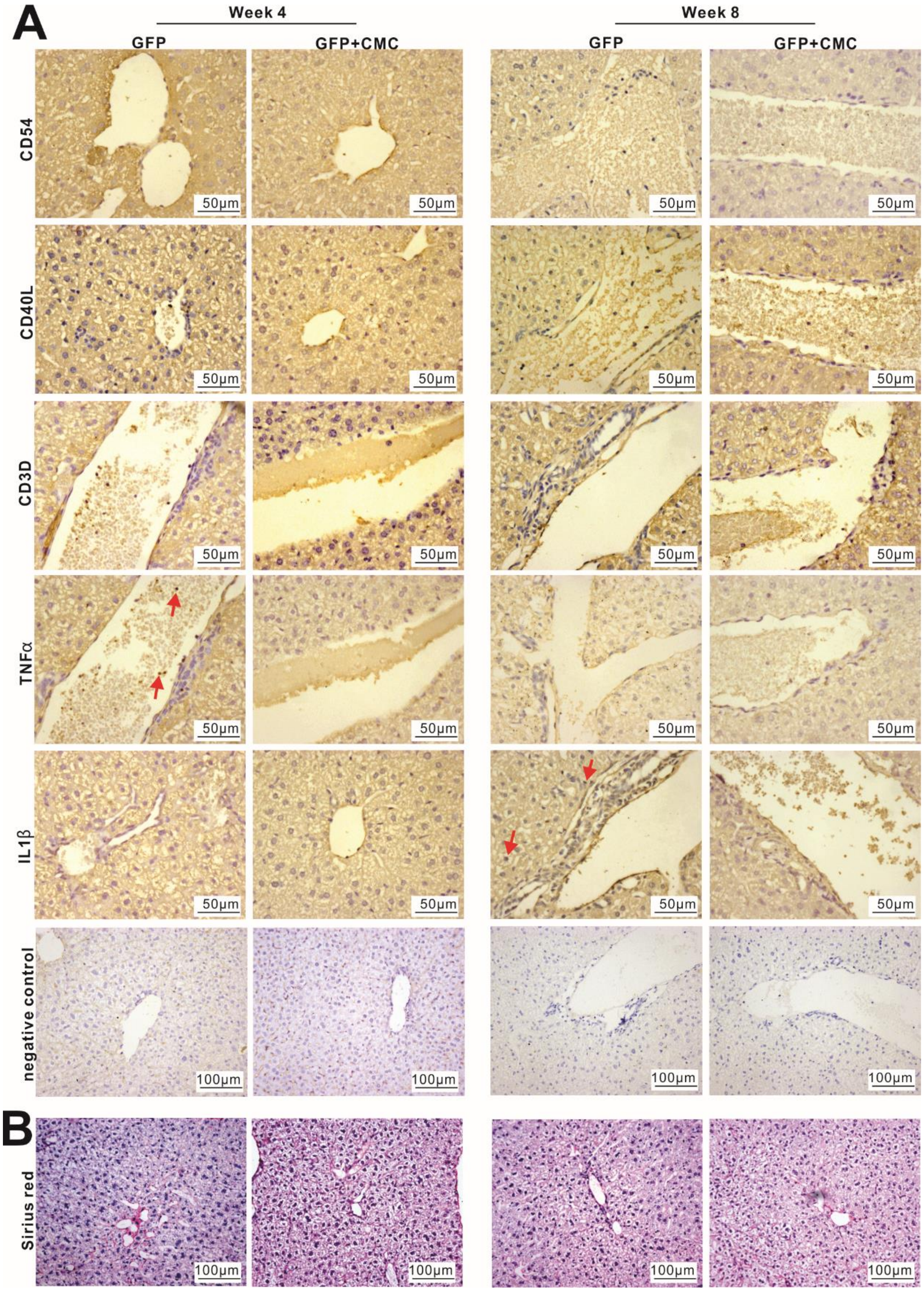

Figure S3. (A) The retrieved liver tissues in **Fig. 4** were subjected to IHC staining with primary antibodies against CD54, CD40L, CD3D, TNFα, and IL1β, or negative control (minus antibody), and representative positive stains are indicated with red arrows. Representative images are shown (x400). **(B)** The retrieved liver samples from weeks 4 and 8, respectively, in **Fig. 4** were subjected to Sirius red staining (x200).

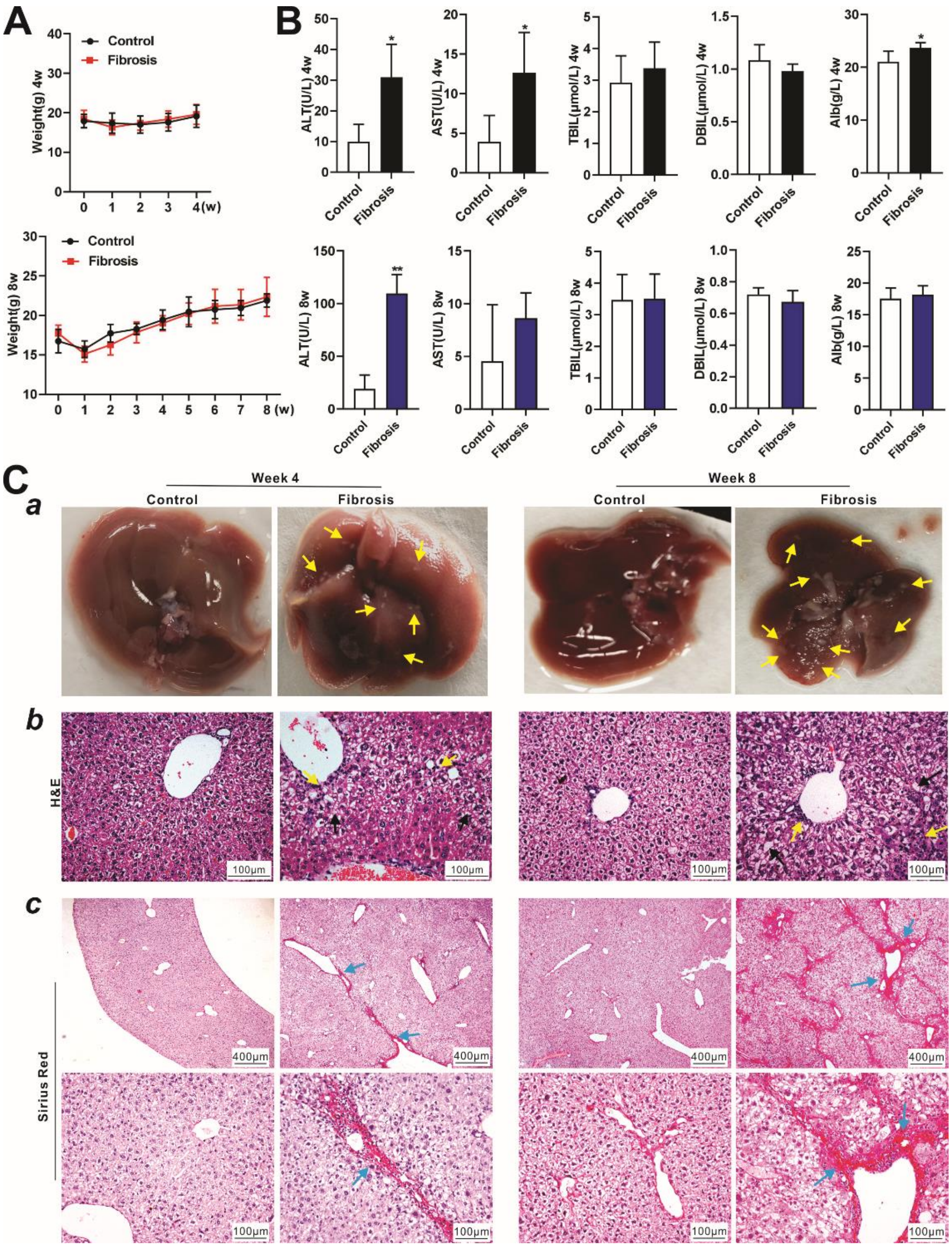

Figure S4. Characterization of the CCl_4-induced liver fibrosis mouse model. Intraperitoneal injection of CCl_4 was used to induce mouse hepatic fibrosis. Mice were sacrificed at weeks 4 and 8, respectively. **(A)** The dynamic weight changes during the course of the experiment. **(B)** The levels of serum ALT, AST, TBIL, DBIL and Alb were measured. "**" $p < 0.01$, "*" $p < 0.05$, Control group vs. Fibrosis group. **(C)** The gross appearance of the retrieved livers was observed, and representative fibrotic nodules are indicated with yellow arrows (***a***). The retrieved liver tissues were subjected to H & E staining (***b***). Representative hepatic fibrotic damage on hepatocytes and necrosis are indicated with black arrows, while representative infiltrating inflammatory cells are indicated by yellow arrows (x200). The retrieved liver tissues were also subjected to Sirius red staining, and the positively stained collagen fibers are indicated with blue arrows (x50 and x200) (***c***). Representative results are shown.

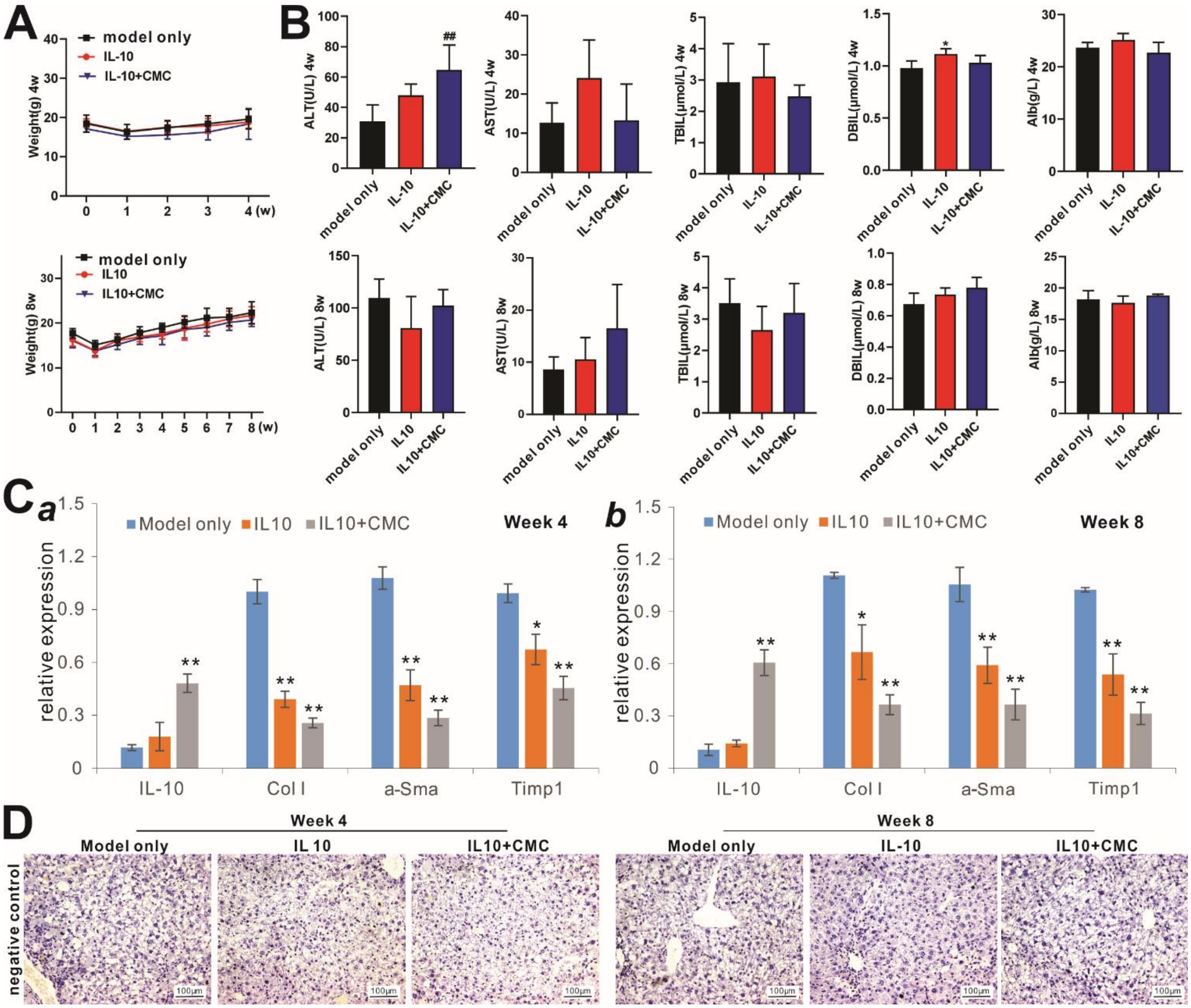

Figure S5. (A) The weekly dynamic weight changes of mice in **Fig. 5** were recorded for week 4 and week 8 groups, respectively. **(B)** The serum levels of ALT, AST, TBIL, DBIL and Alb were determined for week 4 and week 8 groups, respectively. "*" $p < 0.05$, Fibrosis "model only" group vs. IL-10 group. "##" $p < 0.01$, Fibrosis "model only" group vs. IL-10+CMC group. **(C)** Total RNA was extracted from mouse liver tissues. The expressions of IL10 and liver fibrosis related genes was detected by qPCR at week 4 and week 8 respectively. "*" $p < 0.05$, "**" $p < 0.01$, compared with the fibrosis "model only" group. **(D)** IHC negative controls for **Fig. 6**. Paraffin sections of liver samples from week 4 and week 8 groups in **Fig. 5** were subjected to IHC staining without primary antibody (x200). Representative images are shown.
